# Psicosis and suicide risk: who, when and why

**DOI:** 10.1192/j.eurpsy.2022.2182

**Published:** 2022-09-01

**Authors:** J. Gonçalves Cerejeira, I. Santos Carrasco, C. De Andrés Lobo, C. Vallecillo Adame, T. Jiménez Aparicio, G. Guerra Valera, M. Queipo De Llano De La Viuda, A. Gonzaga Ramírez, O. Martin-Santiago

**Affiliations:** 1 Hospital Clínico Universitario de Valladolid, Psychiatry, Valladolid, Spain; 2 Hospital Clínico Universitario de Valladolid, Psiquiatría, VALLADOLID, Spain; 3 Hospital Clínico Universitario, Psiquiatría, Valladolid, Spain; 4 HOSPITAL CLINICO UNIVERSITARIO DE VALLADOLID, Psychiatry, Valladolid, Spain

**Keywords:** Psychosis, Suicide

## Abstract

**Introduction:**

Suicide rates in people diagnosed with a psychotic disorder can be up to 50 times higher than in the general population, with the lethality of attempts being significantly higher in this group, compared to people diagnosed with other psychiatric disorders. Furthermore, it is known that being male is associated with more serious suicide attempts and higher rates of completed suicides.

**Objectives:**

To reflect on the increased risk of suicide associated with psychotic disorders.

**Methods:**

Case report and literature review.

**Results:**

Case report 40-year-old male, recently diagnosed with Schizophreniform Disorder and currently with persistent positive symptoms. He was admitted to our psychiatric hospitalization unit due to a voluntary overdose of almost 100 tablets (antihypertensives, antiarrhythmics, and benzodiazepines) and alcohol. He admits taking the pills with the aim of committing suicide. Literature review: - Around 10% of people diagnosed with schizophrenia commit suicide. - In young patients diagnosed with schizophrenia, suicide is the leading cause of death. - Between 15 and 65% of patients diagnosed with schizophrenia have depressive symptoms such as hopelessness. - Depressive symptoms in these patients seem to be directly proportionally with awareness of the disease (stigma, awareness of its severity and a sudden decrease in quality of life and social integration). - The risk of suicide increases especially in the first 10 years of the disease.

**Conclusions:**

Psychosis is an important risk factor of suicide and active preventive measures should be carried out in these patients.

**Disclosure:**

No significant relationships.

